# Longitudinal Association Between Severe Early Childhood Caries and Mandibular Condylar Trabecular Organization: A 3-Year Retrospective Controlled Study

**DOI:** 10.3390/jcm15166416

**Published:** 2026-08-19

**Authors:** Demet Demir Yıldırım, Ebru Hazar Bodrumlu, Ahmet Yıldırım, Orhan Cicek

**Affiliations:** 1 Department of Pediatric Dentistry, Faculty of Dentistry, Zonguldak Bülent Ecevit University, Zonguldak 67600, Türkiye; hazarebru@yahoo.com; 2 Department of Orthodontics, Faculty of Dentistry, Zonguldak Bülent Ecevit University, Zonguldak 67600, Türkiye; drahmettyildirim@gmail.com (A.Y.); orhancicek@beun.edu.tr (O.C.)

**Keywords:** severe early childhood caries, fractal dimension, mandibular condyle, trabecular bone, panoramic radiography

## Abstract

**Objectives**: The objective of this study was to longitudinally evaluate mandibular condylar trabecular organization in children with a history of severe early childhood caries (SECC) and caries-free controls using fractal dimension (FD) analysis, and to compare the findings according to sex. **Methods**: This retrospective follow-up study included 100 children (50 with a history of SECC and 50 caries-free controls) with panoramic radiographs obtained at T0 (5 years) and T1 (8 years). FD analysis of the mandibular condyle was performed using the box-counting method on standardized regions of interest (ROIs; 21 × 21 pixels) following image calibration. Independent and paired t-tests and a linear mixed-effects model were used for statistical analysis at *p* < 0.05. **Results**: No significant difference in FD values was observed between the SECC and control groups at T0 (*p* > 0.05). At T1, the control group exhibited significantly higher FD values than the SECC group (1.151 ± 0.06 vs. 1.076 ± 0.10, *p* < 0.001). The linear mixed-effects model demonstrated a significant group × time interaction (*p* < 0.001), indicating different longitudinal trajectories between the groups. Within-group comparisons showed no significant longitudinal change in the SECC group (*p* > 0.05), whereas FD values increased significantly in the control group (*p* < 0.001). No significant effects of sex or related interactions were identified (*p* > 0.05). **Conclusions**: Children with a history of SECC did not exhibit the age-related longitudinal increase in mandibular condylar FD values observed in caries-free controls. These findings suggest that a history of SECC may be associated with an altered longitudinal pattern of condylar trabecular organization during growth; however, further studies are required to confirm this association.

## 1. Introduction

Early childhood caries (ECC), an aggressive form of dental caries recognized as a diet-related infectious disease, is among the most prevalent biofilm-mediated chronic multifactorial infectious diseases affecting children worldwide and is associated with numerous biological, physiological, and behavioral risk factors [1,2]. According to the American Academy of Pediatric Dentistry (AAPD), ECC is defined as the presence of one or more decayed lesions, whether cavitated or non-cavitated, in any primary tooth, caries-related tooth loss, or filled tooth surfaces in children aged 72 months or younger. Severe early childhood caries (SECC) is defined as the presence of any carious lesion on a smooth surface in children younger than 3 years of age. In children aged 3, 4, and 5 years, SECC is indicated by the presence of at least 4, 5, or 6 decayed, missing, or filled tooth surfaces, respectively [3]. ECC can lead to adverse effects on growth and development, nutritional disturbances, and impairments in oral health-related quality of life, affecting both children and their families [3,4].

Primary teeth, which play a critical role in a child’s growth and development, not only guide the eruption of permanent teeth and contribute to the establishment of ideal occlusion, but also serve important functions in mastication, esthetics, speech development, and the prevention of deleterious oral habits. When left untreated, premature loss of primary teeth due to ECC may result in uncontrolled movement of adjacent teeth into the edentulous space, potentially leading to malocclusions, while impaired masticatory function may also contribute to reduced masticatory muscle strength [5]. Even when carious teeth are treated, the persistence of cariogenic dietary habits constitutes a substantial risk factor for disease recurrence [6]. Considering these adverse consequences of ECC, elucidating its relationship with growth and developmental processes is of considerable importance; accordingly, structures that play a critical role in craniofacial development should be comprehensively investigated. Because the mandibular condyle undergoes continuous remodeling in response to functional mechanical loading during growth, alterations in masticatory function associated with ECC may influence condylar trabecular adaptation [7].

The mandible, one of the principal components of the craniofacial system, consists of four functional components, including the condyle, ramus, corpus, and alveolus. Understanding mandibular growth requires the separate evaluation of each of these components. Among them, the mandibular condyle is defined as a growth site capable of adapting to changes occurring in other regions of the face [8]. In response to the downward and forward translation of the mandible, the condyle grows upward and backward, accompanied by substantial bone remodeling [9]. Therefore, a comprehensive evaluation of the hard tissue changes that play a determining role in mandibular growth also necessitates detailed investigation of the trabecular bone pattern, which reflects the internal architecture of these structures.

The trabecular bone pattern can be characterized by various structural parameters, including trabecular thickness, trabecular spacing, bone marrow spaces, and bone fractal dimension (FD). The internal architecture of living bone possesses the ability to remodel in response to mechanical loading; therefore, trabecular bone, which exhibits high metabolic activity, is considered an important indicator for the evaluation of alterations in bone architecture [10]. FD analysis, which is used for the quantitative assessment of trabecular organization, is a mathematical method that evaluates irregular and complex structures and provides information regarding trabecular organization [11]. FD values reflect the complexity of the trabecular structure and bone density, with lower values indicating a more porous structure with wider marrow spaces, whereas higher values are associated with a denser and more complex trabecular organization [11,12]. Furthermore, its relative resistance to variations in radiographic density and projection geometry makes FD analysis a useful and objective method for assessing changes in bone structure [12,13,14].

In the literature, FD analysis has been employed for the assessment of midpalatal suture maturation [15], evaluation of mandibular trabecular alterations associated with dental anomalies such as hypodontia and infraocclusion [16,17,18], investigation of trabecular bone characteristics in systemic disorders including osteogenesis imperfecta [19], and analysis of age-related changes in mandibular condylar trabeculation in children [20]. Previous studies have also explored the association between dental caries and mandibular trabecular structure [21]. However, to the best of our knowledge, no longitudinal study has investigated the association between a history of SECC and mandibular condylar trabecular organization. Therefore, the novelty of the present study lies in being the first longitudinal FD assessment of mandibular condylar trabecular organization in children with a history of SECC compared with caries-free controls. The findings may offer clinically relevant insights for both pediatric dentists and orthodontists by highlighting the importance of early diagnosis and management of SECC while providing a basis for future studies investigating its potential association with craniofacial growth and development.

In light of these considerations, the aim of the present study was to longitudinally investigate differences in mandibular condylar trabeculation between children with a history of SECC and caries-free healthy controls over a 3-year retrospective follow-up using FD analysis, and to compare the findings according to sex. The first null hypothesis of the study was that FD values would not differ significantly between the SECC and control groups or over time. The second null hypothesis was that sex would not have a significant effect on FD values between groups or over time.

## 2. Materials and Methods

### 2.1. Study Design, Ethics, and Consent

This retrospective longitudinal observational study, designed as a three-year follow-up, used panoramic radiographs obtained at two predefined time points, T0 (5 years) and T1 (8 years), retrieved from the clinical archives of children with a history of SECC and caries-free healthy children. All participants were referred to the Departments of Pediatric Dentistry and Orthodontics at Zonguldak Bülent Ecevit University, where all panoramic radiographs included in the present study had been obtained during routine pediatric and orthodontic follow-up examinations when clinically indicated.

The Non-Interventional Clinical Research Ethics Committee of Zonguldak Bülent Ecevit University approved the study (decision no. 2026/08-8, 22 April 2026). Written informed consent for the use of anonymized clinical and radiographic data for scientific research was routinely obtained from all patients and/or their legal guardians at the time of treatment. Because of the retrospective nature of the study, no additional study-specific informed consent was required.

### 2.2. Sample Size Calculation, Groups, and Criteria

An a priori power analysis was performed using G*Power software (version 3.1.9.7, Franz Faul, Kiel University, Kiel, Germany) to determine the required sample size. Based on a two-tailed independent-samples *t*-test (difference between two independent means), the analysis was conducted using an effect size of *d* = 0.6889656, derived from a previous study by Bulut and Tokuc [20], with a significance level of α = 0.05, a desired statistical power of 0.90 (1 − *β*), and an allocation ratio of 1:1 between groups. The analysis indicated that a minimum of 92 participants (46 in each group) was required to achieve adequate statistical power (noncentrality parameter δ = 3.3041629, critical *t* = 1.9866745, degrees of freedom [df] = 90, achieved power = 0.9046889). A total of 100 participants (50 per group) were included, exceeding the minimum sample size requirement, with balanced sex distribution between groups to minimize potential confounding effects.

To further evaluate the adequacy of the final sample size for the primary longitudinal analysis, a post hoc sensitivity analysis was additionally performed using a repeated-measures within–between interaction model in G*Power. Using *α* = 0.05, a statistical power of 0.90, two groups, two repeated measurements, a correlation of 0.50 among repeated measurements, and a nonsphericity correction of ε = 1, the final sample size of 100 participants was sufficient to detect a minimum interaction effect size of *f* = 0.164.

The study population was divided into two groups: the SECC group and the control group. Each group was further subdivided by sex, with an equal number of males and females in each group (25 males and 25 females). At T0, the mean age was 5.31 ± 0.32 years in the SECC group and 5.23 ± 0.19 years in the control group, whereas at T1, the mean age was 8.19 ± 0.17 years and 8.28 ± 0.26 years, respectively. There were no statistically significant differences between the groups in terms of age at either time point (*p* > 0.05).

Children were assigned to the SECC group based on baseline clinical records documenting a previous diagnosis of SECC, established according to the age-specific diagnostic criteria of the American Academy of Pediatric Dentistry (AAPD) [3] by two experienced pediatric dentists (D.D.Y. and E.H.B.). All children had completed the comprehensive dental treatment considered clinically necessary before the T0 radiographic examination.

Inclusion criteria for the SECC group:

- Children aged 5 years with a history of SECC;
- Completion of comprehensive SECC treatment and attendance at clinical follow-up examination at T1;
- Availability of high-quality, high-resolution panoramic radiographs in Tag Image File Format (TIFF) at both T0 and T1.

Inclusion criteria for the control group:

- Children with no history of dental caries, caries-related tooth loss, or dental restorations who underwent clinical examination at both T0 and T1;
- Availability of high-quality, high-resolution panoramic radiographs in TIFF at both T0 and T1.

Exclusion criteria (for both groups):

- Presence of craniofacial anomalies, systemic bone diseases, or syndromes affecting growth and development;
- History of orthodontic treatment, surgical intervention, or trauma affecting mandibular development;
- Absence of at least one panoramic radiograph from the T0 or T1 periods;
- Panoramic radiographs of insufficient quality or with distortion preventing accurate evaluation.

### 2.3. Clinical Interventions for SECC During the Follow-Up Period

For this study, a total of 1513 patient records in the archive were evaluated, and 100 children who met the inclusion criteria were included in the study. All treatments provided to children in the SECC group following the diagnosis of SECC were performed at the Department of Pediatric Dentistry, Faculty of Dentistry, Zonguldak Bülent Ecevit University, according to the treatment needs of each patient and the recommendations of the American Academy of Pediatric Dentistry (AAPD).

Within the SECC group, restorative and endodontic treatments (including pulpotomy, root canal treatment, and apexification) were performed in 14 children (28%), whereas 36 children (72%) received these treatments in addition to prosthetic treatment, tooth extraction, and space maintainer therapy when clinically indicated. No orthodontic treatment or surgical intervention affecting mandibular growth or trabecular bone structure was performed during the study period.

### 2.4. Radiographic Acquisition and FD Analysis

Panoramic radiographs were obtained at T0 and T1 using the same X-ray unit (Veraviewepocs 2D, J. Morita Mfg. Corp., Kyoto, Japan) under standardized institutional positioning protocols, with the Frankfurt horizontal plane aligned parallel to the floor and exposure parameters of 70 kVp and 7.5 mA. All radiographs were retrieved in TIFF, and only those with sufficient image quality and resolution were included in the analysis.

FD analysis was performed using ImageJ software (version 1.53, National Institutes of Health, Bethesda, MD, USA), based on the box-counting method described by White and Rudolph [9]. Before FD analysis, all radiographic images were anonymized by removing patient identifiers and were evaluated in a randomized order. All image processing and analyses were then conducted by a single experienced investigator (O.C.) with extensive experience in FD analysis [22,23,24,25], using the same computer system (ASUS TUF Gaming F15 FX507ZC4_FX507ZC4, ASUSTeK Computer Inc., Taipei, Taiwan) to ensure methodological consistency. To minimize potential bias, the investigator was blinded to group allocation during image processing and FD measurement.

Prior to each FD analysis, image calibration was performed using the *Set Scale* function in ImageJ based on a pixel size of 0.096 mm to ensure measurement standardization across all radiographs. After calibration, square regions of interest (ROIs) measuring 21 × 21 pixels (approximately 2.0 × 2.0 mm) were defined in the right mandibular condyle for all subjects. Considering the relatively small dimensions of the mandibular condyle in this pediatric population, this ROI size was considered appropriate for including a representative region of the condylar trabecular bone, consistent with the standardized ROI dimensions previously used in FD studies evaluating mandibular trabecular bone [11,17,18]. The selected ROI was designed to minimize the influence of cortical bone, anatomical superimposition, and image artifacts. ROIs were consistently positioned within the central trabecular region of the right mandibular condyle while avoiding the cortical outline, articular surface, and overlapping anatomical structures. A preliminary bilateral comparison was performed using radiographs from 25 randomly selected participants (25% of the total sample), including children from both the SECC and control groups. Bilateral FD measurements were evaluated at both T0 and T1 using a paired-samples t-test. No statistically significant bilateral difference was detected (*p* = 0.892); therefore, only the right condyle was included in the final analysis to standardize measurements and avoid statistical dependence between bilateral observations.

**Figure 1:**
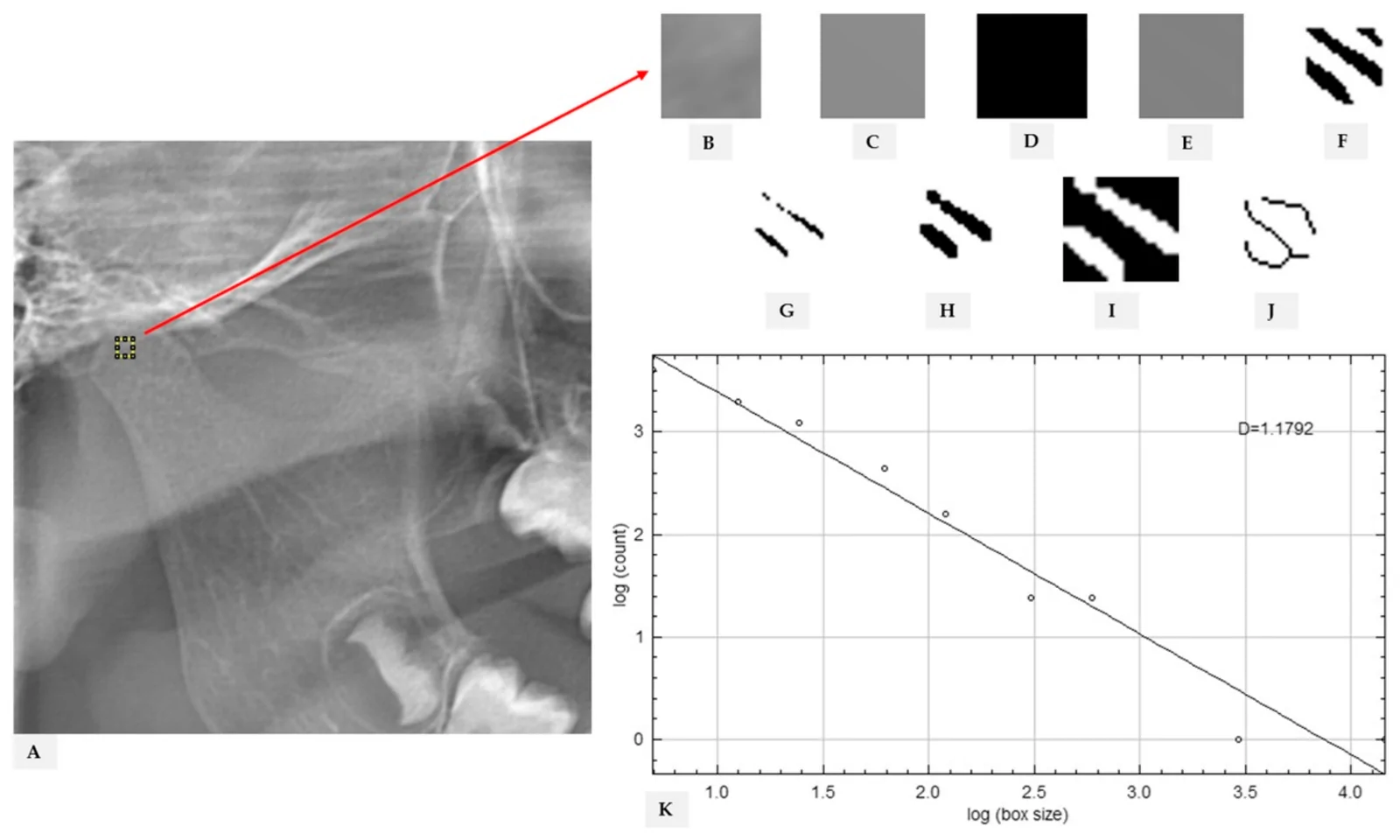
Step-by-step illustration of the FD analysis procedure applied to the right mandibular condyle. (**A**) Standardized selection of the region of interest (ROI; 21 × 21 pixels) within the central trabecular region of the right mandibular condyle, with the red arrow indicating the transition from the selected ROI to the duplicated image, illustrating the first step of the FD analysis. (**B**) Duplication of the ROI. (**C**) Application of Gaussian blur (sigma = 35). (**D**) Image subtraction. (**E**) Addition of a constant gray value (128). (**F**) Conversion to binary image (mask). (**G**) Erosion. (**H**) Dilation. (**I**) Inversion. (**J**) Skeletonization of the trabecular pattern. (**K**) Box-counting analysis used to calculate the FD value.

Each selected ROI was converted to 8-bit format, and a Gaussian blur filter (sigma = 35 pixels) was applied to reduce brightness variations and soft tissue noise. The blurred image was subtracted from the original image, and the result was converted into a binary image by adding a gray value of 128. To further reduce noise, a sequence of image processing steps—erosion, dilation, and inversion—was applied. The processed image was then skeletonized to obtain the trabecular skeleton for fractal analysis, following the standard ImageJ box-counting protocol described by White and Rudolph [10] and subsequently adopted in previous FD studies [11,17,18,22]. Finally, FD values were calculated from the skeletonized images using the box-counting method. The steps of the FD analysis are illustrated in Figure 1.

### 2.5. Measurement Error and Reliability

To assess intra-observer reliability, repeated FD measurements of the right mandibular condyle were obtained from 25 randomly selected participants (25% of the total sample) by the same examiner (O.C.) after a two-week interval. The magnitude of measurement error was quantified using Dahlberg’s formula [26,27]:

$$E=\sqrt{\frac{\sum d^{2}}{2n}}$$

where *E* denotes the measurement error, *d* represents the difference between repeated measurements, and *n* corresponds to the number of observations. Furthermore, intra-observer reliability was evaluated using the intraclass correlation coefficient (ICC) based on a two-way random-effects model with a consistency definition. ICC values and their 95% confidence intervals were calculated to assess the degree of agreement between repeated measurements.

### 2.6. Statistical Analysis

Statistical analyses were performed using SPSS Statistics software (version 26.0; IBM Corp., Armonk, NY, USA). The normality of data distribution was assessed using the Kolmogorov–Smirnov and Shapiro–Wilk tests. As the data were normally distributed, parametric methods were applied. A *p*-value < 0.05 was considered statistically significant.

The primary inferential analysis was conducted using a linear mixed-effects model to evaluate the effects of time, group, sex, and their interactions on FD values while accounting for the repeated-measures structure of the data. Subjects were included as a random effect, and time, group, sex, and their interactions were specified as fixed effects. Model results were reported as fixed-effect estimates (*β* coefficients), standard errors (SEs), 95% confidence intervals (95% CIs), and Type III tests of fixed effects. Estimated marginal means, corresponding mean differences, and 95% confidence intervals were calculated to facilitate interpretation of the longitudinal changes.

Independent-samples *t*-tests were performed as supplementary pairwise comparisons for between-group and sex-based comparisons at individual time points, whereas paired-samples *t*-tests were used as supplementary within-group comparisons over time. Directional change was calculated as the arithmetic difference between T1 and T0 (T1 − T0), whereas absolute individual change was calculated as the absolute value of the individual difference (|T1 − T0|). Effect sizes were calculated using Hedges’ *g* for independent comparisons and Cohen’s *dz* for paired comparisons.

## 3. Results

Table 1 summarizes the intra-observer reliability and measurement error of FD measurements. Measurement error assessed using Dahlberg’s formula was low across all measurements, ranging from 0.026 to 0.044. Intra-observer reliability analysis demonstrated high consistency of repeated measurements, with ICC values indicating strong agreement across both groups and time points. At T0, ICC values were 0.92 (95% CI: 0.68–0.98) for the SECC group and 0.93 (95% CI: 0.70–0.98) for the control group, whereas at T1, ICC values were 0.94 (95% CI: 0.77–0.99) and 0.89 (95% CI: 0.54–0.97), respectively.

Table 2 presents the results of the linear mixed-effects model evaluating the effects of group, time, sex, and their interactions on FD values. A significant main effect of time was observed (*p* = 0.041), whereas the main effects of group (*p* = 0.109) and sex (*p* = 0.208) were not statistically significant. A significant group × time interaction was identified (*p* < 0.001), indicating that the longitudinal pattern of FD changes differed between the SECC and control groups. No significant interactions were found for group × sex (*p* = 0.679), sex × time (*p* = 0.376), or group × sex × time (*p* = 0.509).

Table 3 presents the estimated marginal means of FD values derived from the linear mixed-effects model. The model-based estimates demonstrated an increase in FD values over time in the control group, whereas the SECC group exhibited a slight decrease. These findings were consistent with the significant group × time interaction identified in the linear mixed-effects model, indicating distinct longitudinal trajectories of condylar trabecular organization between the two groups. Based on the estimated marginal means, the adjusted mean difference between T1 and T0 was 0.025 (95% CI: 0.001 to 0.048; *p* = 0.041), whereas the adjusted mean difference between the control and SECC groups was 0.022 (95% CI: −0.005 to 0.050; *p* = 0.109).

Supplementary pairwise comparisons (Table 4) were consistent with the results of the linear mixed-effects model. No statistically significant difference in FD values was observed between the SECC and control groups at T0, whereas the control group demonstrated significantly higher FD values than the SECC group at T1, with a large effect size. Similarly, no significant longitudinal change in FD values was observed within the SECC group, whereas the control group exhibited a significant increase over time with a moderate-to-large effect size.

The mean directional change in FD (T1 − T0) was positive in the control group (95% CI: 0.047 to 0.109) and slightly negative in the SECC group (95% CI: −0.065 to 0.007). Table 4 also summarizes the absolute individual change (|T1 − T0|), which reflects the magnitude of change irrespective of its direction. Although no significant between-group difference was observed in absolute individual change, the linear mixed-effects model demonstrated a significant group × time interaction, indicating distinct longitudinal trajectories between the two groups.

Table 5 summarizes the sex-based comparisons of FD values between groups and across time points. At T0, no statistically significant differences were observed between the SECC and control groups or between females and males within either group. At T1, the control group exhibited significantly higher FD values than the SECC group in both females and males, whereas no significant sex-based differences were detected within either group. Longitudinal analyses showed no significant change in FD values over time among females or males in the SECC group. In contrast, both female and male participants in the control group demonstrated a significant increase in FD values from T0 to T1.

**Figure 2:**
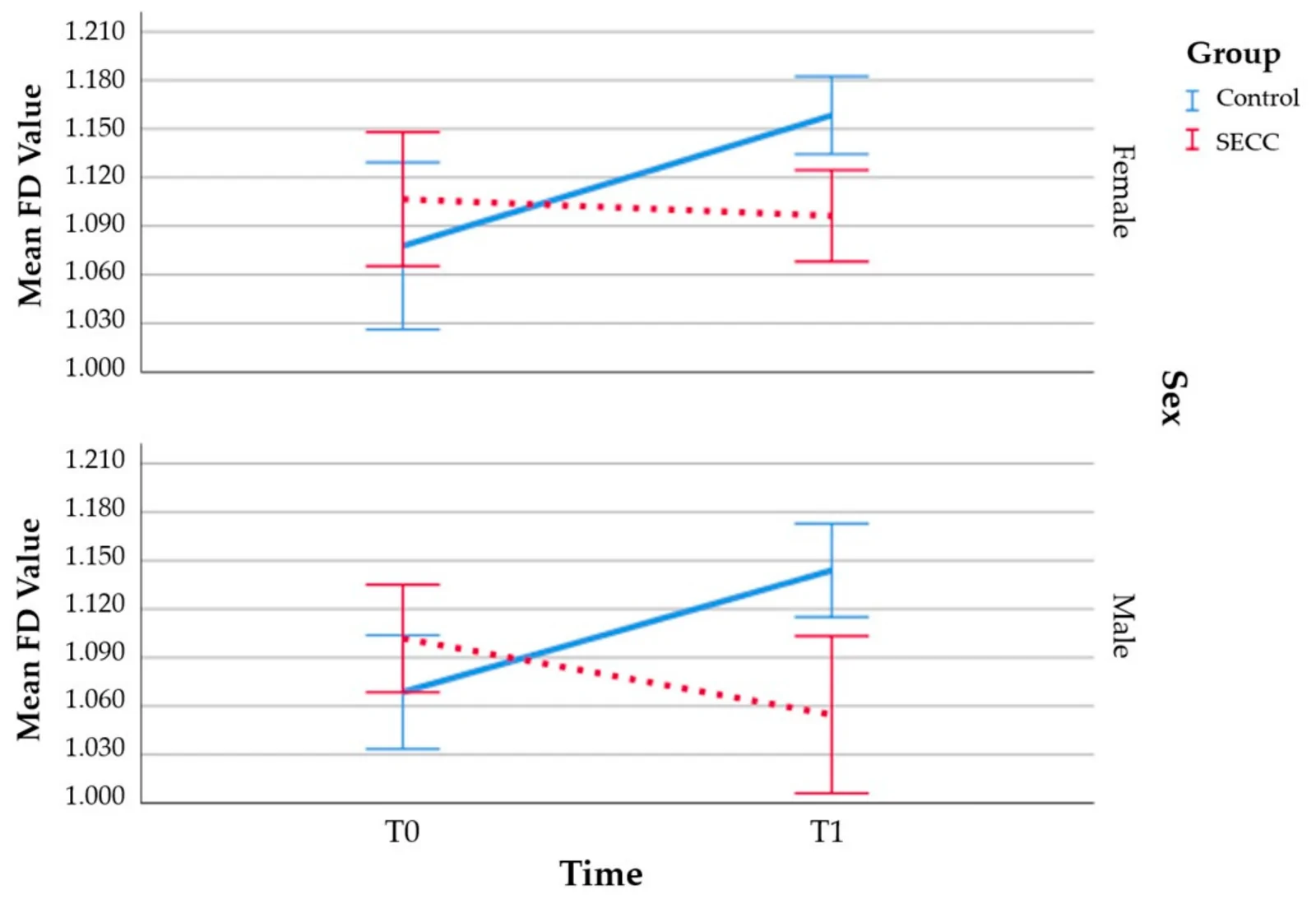
Longitudinal changes in FD values from T0 to T1 in the SECC and control groups according to sex (mean ± 95% CI). FD: fractal dimension; SECC: severe early childhood caries. The blue solid line represents the change in FD values from T0 to T1 in the control group, whereas the red dashed line represents the corresponding change in the SECC group.

As illustrated in Figure 2, the longitudinal trajectories of FD values differed between the SECC and control groups. No apparent between-group difference was observed at T0, whereas the trajectories diverged at T1, with the control group exhibiting higher mean FD values than the SECC group. Over time, mean FD values increased in the control group, whereas they remained relatively stable or showed a slight decrease in the SECC group. This pattern was similar in both females and males. These visual trends were consistent with the significant group × time interaction identified in the linear mixed-effects model (*p* < 0.001), whereas the similar patterns across sexes were consistent with the absence of a significant group × sex × time interaction.

## 4. Discussion

In the present study, the age-related increase in FD values observed in mandibular condylar trabeculation during growth in healthy children was not similarly evident in the SECC group. Although no significant difference was observed between the groups at T0, FD values increased significantly in the control group at T1, whereas a non-significant decreasing trend was identified in the SECC group. Furthermore, the significantly higher FD values detected in the control group compared with the SECC group at T1, together with the significant group × time interaction identified in the linear mixed-effects model analysis, resulted in rejection of the first null hypothesis. The non-significant difference in absolute individual change does not contradict the significant group × time interaction, because absolute values quantify only the magnitude of change, whereas the mixed-effects model captures the direction of the longitudinal trajectories. In contrast, the absence of a significant main effect of sex on FD values, as well as the lack of significant interactions involving sex, group, and time, supported acceptance of the second null hypothesis. The present findings suggest that children with a history of SECC exhibited a distinct pattern of age-related changes in condylar trabecular organization compared with caries-free controls.

The ability of living bone to adapt its internal architecture in response to mechanical loading represents one of its fundamental biological properties. Trabecular bone architecture exhibits a dynamic structure optimized for load-bearing function and undergoes continuous remodeling in response to applied mechanical forces [10]. Because the density and complexity of trabecular organization may directly influence orthopedic and orthodontic processes, including the efficiency of tooth movement, magnitude of applied force, and treatment duration, comprehensive evaluation of bone microarchitecture may contribute to the development of individualized treatment strategies [27,28]. Although several methods have been used to assess trabecular bone structure, including micro-computed tomography (micro-CT) [29], magnetic resonance imaging (MRI) [30], histomorphometry [31], dual-energy X-ray absorptiometry (DEXA) [32], intensity-based imaging features [33], and finite element analysis (FEA) [34], FD analysis has been widely preferred because it enables quantitative assessment of radiographic trabecular complexity using existing panoramic radiographs without the need for additional imaging procedures [24,35].

FD analysis of mandibular bone trabeculation using panoramic radiographs represents a quantitative approach widely utilized across various dental disciplines to evaluate the effects of dental and developmental conditions on jaw bone structure in pediatric populations [22,35,36,37]. In children with molar–incisor hypomineralization, no significant differences in FD values reflecting trabecular bone structure have been reported compared with healthy controls, although reductions in cortical bone parameters have been observed [38]. Similarly, the presence of bruxism in children and adolescents has been associated with increased FD values in specific mandibular regions [39], whereas lower FD values in the mandibular molar region have been documented in preterm and low-birth-weight children [40]. Nevertheless, to the best of our knowledge, no previous study has investigated the association between a history of SECC and condylar trabeculation using FD analysis over a 3-year follow-up period in comparison with caries-free healthy children and according to sex. In this regard, the present study is distinguished by its retrospective, longitudinal, and quantitative design, providing novel insight into the association between a history of SECC and condylar trabecular organization during childhood growth and development.

Bulut and Tokuç [20] reported an age-related increase in FD values in their evaluation of mandibular condylar trabecular structure in children aged 6–13 years. Similarly, Arslan et al. [41] demonstrated that mean condylar FD values increased progressively with age. Consistent with these findings, the present study demonstrated a significant age-related increase in FD values within the control group. In contrast, the SECC group exhibited a non-significant decreasing trend, in contrast to the significant increase observed in healthy controls. Moreover, although comparable FD values were identified between the groups at T0, lower FD values were observed in the SECC group relative to the control group at T1. Although no significant between-group difference was observed in the absolute individual change in FD values, the linear mixed-effects model demonstrated a significant group × time interaction, indicating different longitudinal trajectories of condylar trabecular development between the two groups. These findings suggest that children with a history of SECC may exhibit a more limited age-related progression of condylar trabecular organization, with the physiological increase observed in healthy children appearing to be attenuated.

Children’s trabecular bone density is considered an important biological marker in orthodontic and orthopedic treatment planning [27,28,37]. It has been suggested that individuals with denser trabecular architecture may require greater force to achieve tooth movement, while individual variations in trabecular organization may influence both treatment duration and the magnitude of applied force [28]. In this context, the present findings suggest that a history of SECC should not be regarded solely as a localized oral disease affecting dental hard tissues, but rather as a condition that may be associated with long-term alterations in craniofacial growth patterns. In particular, the differences observed in condylar trabecular organization indicate that oral health status during childhood may be associated with the biological trajectory of craniofacial development.

Functional orthopedic appliances have been reported to influence the direction of condylar growth and to induce alterations in mandibular trabeculation through bone remodeling processes occurring in specific regions of the mandible [42]. Cesur et al. [37] demonstrated a positive correlation between right condylar FD values and ramus height, suggesting that appliance-induced modifications in condylar bone structure may be associated with mandibular growth dynamics. Within this context, the lower condylar FD values observed in the SECC group may reflect altered patterns of condylar adaptation and trabecular remodeling during the growth period.

Although the present study did not directly evaluate functional parameters, the observed radiographic findings may be interpreted in the context of biological mechanisms proposed in previous experimental and clinical studies [43,44]. Pain, cavitation, posterior support loss, and reduced masticatory efficiency associated with SECC may alter masticatory function in children, potentially leading to changes in masticatory muscle activity and mandibular loading patterns. Almotairy et al. [45] reported that food hardness can influence chewing behavior and jaw muscle adaptation in children, thereby modifying masticatory patterns. Accordingly, childhood caries has been associated with impaired masticatory performance, reduced maximum bite force, altered swallowing threshold and chewing behavior, as well as delayed dental development [46,47]. Mustuloğlu et al. [48] demonstrated that both masticatory function and tolerance to food texture were adversely affected in children with SECC. Similarly, Linas et al. [49] reported decreased chewing frequency and the development of compensatory masticatory behaviors in affected children. Improvements in masticatory capacity following comprehensive pediatric dental treatment have also been documented [50].

Within this framework, the absence of the physiological increase in condylar FD values observed in the control group among children with SECC may suggest altered functional loading patterns in the condylar region. Whereas the age-related increase in condylar FD values in healthy children may be regarded as an indicator of normal functional adaptation [20,41], the lower FD pattern identified in the SECC group may be consistent with altered adaptation to physiological loading during growth. These findings suggest that a history of SECC may be associated not only with localized dental destruction but also with differences in mandibular growth dynamics and functional skeletal adaptation. Our findings further support the importance of early pediatric dental intervention and, when indicated, multidisciplinary diagnosis and treatment planning supported by orthodontic evaluation in children with SECC [48,50].

Bone development is a complex process influenced by numerous biological and environmental factors, including age, sex, hormonal status, nutrition, and lifestyle [51]. In addition, skeletal development has been reported to be closely associated with occlusal loading, the number of teeth present, and particularly the presence of posterior dentition [52,53]. Evidence demonstrating increased FD values in the periapical region following Mineral Trioxide Aggregate (MTA) apexification and regenerative endodontic treatment in immature permanent teeth with trauma-induced pulp necrosis further supports the dynamic and treatment-responsive nature of trabecular bone organization [54]. From this perspective, timely diagnosis and management of SECC may be important not only for preserving dental hard tissues, but also for maintaining the physiological remodeling environment necessary for normal condylar development during craniofacial growth.

Although the observed findings are biologically plausible and clinically relevant, they should be interpreted within the methodological constraints of the present study. Furthermore, although the longitudinal design enabled the evaluation of temporal changes in condylar trabecular organization, the retrospective observational nature of the present study does not permit inference of a direct causal relationship between a history of SECC and the observed differences in FD trajectories. The age- and sex-matched study groups and repeated assessments over time strengthen the validity of the observed longitudinal patterns; however, the potential influence of residual confounding factors, including nutritional status, dietary habits, socioeconomic characteristics, oral hygiene behaviors, and other environmental variables, cannot be completely excluded. Similarly, information regarding posterior occlusal support, premature tooth loss, treatment subgroups, body mass index, and systemic medication use was not consistently available because of the retrospective design and therefore could not be incorporated into additional subgroup or sensitivity analyses. Therefore, the present findings should be interpreted as demonstrating an association between a history of SECC and altered longitudinal patterns of condylar trabecular organization rather than establishing a direct causal effect.

This study provides important insights as one of the few longitudinal investigations exploring condylar trabecular organization in children with a history of SECC compared with caries-free controls. Nevertheless, several limitations should be acknowledged, including the assessment of trabecular structure using two-dimensional panoramic radiographs, which are inherently affected by image magnification, distortion, superimposition, and head positioning [55]. Although standardized imaging and ROI selection procedures were applied, these factors may have influenced FD measurements. In addition, the inability to directly evaluate masticatory muscle activity, occlusal forces, functional loading, and other clinical factors potentially related to occlusal function limited interpretation of the underlying biological mechanisms. Despite these limitations, the age- and sex-matched control group, the 3-year follow-up design, the longitudinal FD assessment performed in the same individuals, and the use of a linear mixed-effects model represent important methodological strengths of the study. Future prospective longitudinal studies incorporating three-dimensional imaging modalities such as CBCT, together with functional assessments including masticatory performance, muscle activity, and orthopedic treatment response, may provide a more comprehensive understanding of how a history of SECC relates to mandibular growth and condylar adaptation.

## 5. Conclusions

The findings of this study showed that the age-related increase in trabecular FD values observed in the mandibular condyle during growth in healthy children was not similarly observed in children with a history of SECC. The results suggest that FD analysis may serve as a useful quantitative method for evaluating condylar trabecular structure in growing children. Furthermore, a history of SECC was associated with a different pattern of developmental changes in condylar trabecular organization, which became more pronounced over time. Therefore, early diagnostic and therapeutic approaches for SECC may be important not only for caries control but also when considering normal condylar development during childhood growth. Further prospective studies are warranted to confirm these findings and further clarify the relationship between SECC and condylar development.

## Figures and Tables

**Table 1:** Intra-observer reliability and measurement error of FD measurements at two time points (T0 and T1).

Time	Group	ICC (95% CI)	Dahlberg Error
T0	SECC	0.92 (0.68–0.98)	0.044
	Control	0.93 (0.70–0.98)	0.039
T1	SECC	0.94 (0.77–0.99)	0.041
	Control	0.89 (0.54–0.97)	0.026

SECC: severe early childhood caries, FD: fractal dimension, T0: 5 years, T1: 8 years, ICC: intraclass correlation coefficient, CI: confidence interval. Intra-observer reliability was assessed using repeated FD measurements from 25 randomly selected participants (25% of the total sample).

**Table 2:** Results of the linear mixed-effects model evaluating the effects of group, time, sex, and their interactions on fractal dimension (FD) values.

Effect	*β* (Estimate)	SE	95% CI	*F* (1,96)	*p*
Group	0.089	0.023	0.043 to 0.135	2.614	0.109
Sex	0.042	0.023	−0.004 to 0.088	1.610	0.208
Time	0.047	0.024	0.000 to 0.094	4.305	0.041 *
Group × Sex	−0.027	0.033	−0.092 to 0.038	0.172	0.679
Group × Time	−0.122	0.034	−0.189 to −0.056	20.192	<0.001 *
Sex × Time	−0.037	0.034	−0.103 to 0.030	0.792	0.376
Group × Sex × Time	0.031	0.047	−0.063 to 0.126	0.439	0.509

*β*: fixed-effect regression coefficient; SE: standard error; CI: confidence interval. *β* coefficients, standard errors, and 95% confidence intervals represent parameter estimates relative to the selected reference categories. *F* statistics and corresponding *p* values are derived from Type III tests of fixed effects evaluating the overall significance of each model term (denominator df = 96). Accordingly, the CIs for the *β* estimates are not expected to correspond directly to the *p* values derived from the Type III tests. * *p* < 0.05 indicates statistical significance.

**Table 3:** Estimated marginal means of FD values according to group and time derived from the linear mixed-effects model.

Group	Time	Estimated Marginal Mean	SE	95% CI
Control	T0	1.073	0.014	1.045–1.101
Control	T1	1.151	0.012	1.128–1.174
SECC	T0	1.104	0.014	1.076–1.132
SECC	T1	1.076	0.012	1.053–1.098

FD: fractal dimension; SE: standard error; CI: confidence interval; SECC: severe early childhood caries. Estimated marginal means were obtained from the linear mixed-effects model and are adjusted for the fixed effects included in the model.

**Table 4:** Comparison of FD values between SECC and control groups at T0 and T1.

	SECC Group	Control Group	*p*	Effect Size
	Mean ± SD (Min–Max)	Mean ± SD (Min–Max)		
T0	1.104 ± 0.09 (0.919–1.296)	1.073 ± 0.11 (0.816–1.264)	0.118 ^†^	*g* = 0.31
T1	1.076 ± 0.1 (0.719–1.225)	1.151 ± 0.06 (1.031–1.261)	<0.001 *^,†^	*g* = −0.91
Within-group *p*	0.117 ^P^	<0.001 *^,P^		
Within-group effect size	*dz* = 0.23	*dz* = 0.72		
Absolute individual change in FD (|T1−T0|)	0.095 ± 0.09 (0.004–0.424)	0.102 ± 0.09 (0.001–0.376)	0.673 ^†^	*g* = −0.08

SECC: severe early childhood caries, T0: 5 years, T1: 8 years, FD: fractal dimension, ^†^: independent-samples *t*-test, ^P^: paired samples *t*-test, *p*: significance value, *: *p* < 0.05. Effect sizes were calculated using *Hedges’ g* for independent-samples *t*-tests and *Cohen’s dz* for paired samples *t*-tests.

**Table 5:** Sex-based comparison of FD values between groups and over time (T0–T1).

**Time**	**Sex**	**SECC Group** **Mean ± SD** **(Min–Max)**	**Control Group** **Mean ± SD** **(Min–Max)**	***p***
T0	Female	1.107 ± 0.1 (0.919–1.296)	1.078 ± 0.12 (0.816–1.264)	0.372 ^†^
	Male	1.102 ± 0.08 (0.933–1.243)	1.069 ± 0.09 (0.869–1.179)	0.164 ^†^
	*p* (between sexes)	0.853 ^†^	0.765 ^†^	
T1	Female	1.1 ± 0.07 (0.946–1.225)	1.158 ± 0.06 (1.057–1.261)	0.001 *^,†^
	Male	1.055 ± 0.12 (0.719–1.193)	1.144 ± 0.07 (1.031–1.257)	0.002 *^,†^
	*p* (between sexes)	0.133 ^†^	0.430 ^†^	
**Groups**	**Sex**	**T0** **Mean ± SD** **(Min–Max)**	**T1** **Mean ± SD** **(Min–Max)**	***p***
SECC Group	Female	1.107 ± 0.1 (0.919–1.296)	1.1 ± 0.07 (0.946–1.225)	0.703 ^P^
	Male	1.102 ± 0.08 (0.933–1.243)	1.055 ± 0.12 (0.719–1.193)	0.062 ^P^
Control Group	Female	1.078 ± 0.12 (0.816–1.264)	1.158 ± 0.06 (1.057–1.261)	0.003 *^,P^
	Male	1.069 ± 0.09 (0.869–1.179)	1.144 ± 0.07 (1.031–1.257)	0.001 *^,P^

SECC: severe early childhood caries, T0: 5 years, T1: 8 years, FD: fractal dimension, ^†^: independent-samples *t*-test, ^P^: paired samples *t*-test, *p*: significance value, *: *p* < 0.05.

## Data Availability

The datasets used and/or analysed during the current study are available from the corresponding author on reasonable request.

## Abbreviations

The following abbreviations are used in this manuscript:

ECC	Early childhood caries
SECC	Severe early childhood caries
T0	5 years
T1	8 years
FD	Fractal dimension
ROI	Regions of Interest
CBCT	Cone–beam computed tomography
TIFF	Tag image file format
MRI	Magnetic resonance imaging
DEXA	Dual-energy X-ray absorptiometry
FEA	Finite element analysis
ICC	Intraclass correlation coefficient
CI	Confidence interval
